# 3-(2-Amino-1-methyl-4-oxo-4,5-dihydro-1*H*-imidazol-5-yl)-3-hydr­oxy-1-phenyl­indolin-2-one ethanol solvate

**DOI:** 10.1107/S1600536809033881

**Published:** 2009-09-12

**Authors:** Narsimha Reddy Penthala, Thirupathi Reddy Yerram Reddy, Sean Parkin, Peter A. Crooks

**Affiliations:** a Department of Pharmaceutical Sciences, College of Pharmacy, University of Kentucky, Lexington, KY 40536, USA; b Department of Chemistry, University of Kentucky, Lexington, KY 40506, USA

## Abstract

In the title compound, C_18_H_16_N_4_O_3_·C_2_H_5_OH, mol­ecules are linked into chains by a series of inter­molecular N—H⋯O, N—H⋯N and O—H⋯O hydrogen bonds which stabilize the crystal structure. The indole and creatinine units make a dihedral angle of 56.45 (4)°. The title compound has two chiral centres. The crystal structure indicates the compound is racemic (*RR* and *SS*).

## Related literature

For the biological activity of isatin and its derivatives, see: Pandeya *et al.* (2005 ▶). The endogenous oxindoles 5-hydroxy­oxindole and isatin are anti­proliferative and proapoptotic, see: Cane *et al.* (2000 ▶). For *in vitro* cytotoxicity evaluation of some substituted isatin derivatives, see: Vine *et al.* (2007 ▶). 2-Indol-3-yl-methyl­enequinuclidin-3-ols and NADPH oxidase activity has been reported by Sekhar *et al.* (2003 ▶), and novel substituted (*Z*)-2-(*N*-benzyl­indol-3-ylmethyl­ene)quinuclidin-3-one and (*Z*)-(±)-2-(*N*-benzyl­indol-3-yl methyl­ene)quinuclidin-3-ol derivatives as potent thermal sensitizing agents by Sonar *et al.* (2007 ▶). For the crystal and mol­ecular structure of isatin, see: Frolova *et al.* (1988 ▶). For the structure of 3-(2-amino-1-methyl-4-oxo-4,5-dihydro-1*H*-imidazol-5-yl)-3-hydroxy­indolin-2-one monohydrate, see: Penthala *et al.* (2009 ▶) and of 1,1′-diacetyl-3-hydr­oxy-2,2′,3,3′-tetra­hydro-3,3′-bi(1*H*-indole)-2,2′-dione, see: Usman *et al.* (2002 ▶). The aldol condensation enolate mechanism *via* a six-membered transition state has been described by Zimmerman & Traxler (1957 ▶).
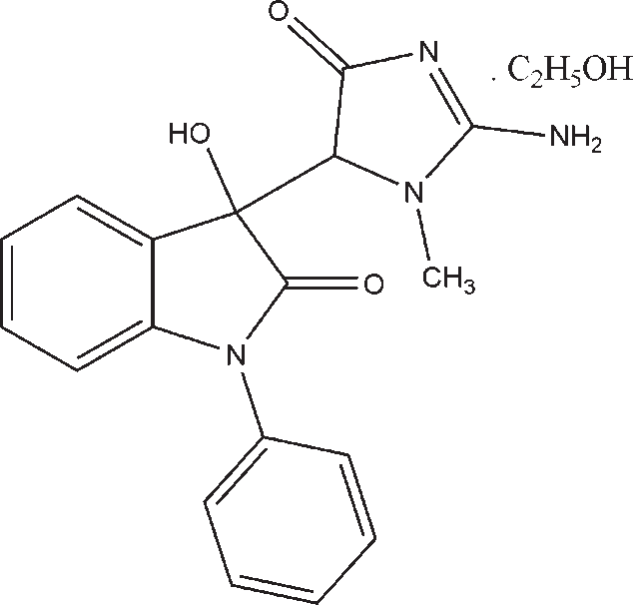


## Experimental

### 

#### Crystal data

C_18_H_16_N_4_O_3_·C_2_H_6_O
*M* *_r_* = 382.42
Triclinic, 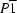

*a* = 7.4912 (1) Å
*b* = 11.0018 (2) Å
*c* = 12.0835 (2) Å
α = 78.152 (1)°
β = 74.413 (1)°
γ = 74.090 (1)°
*V* = 913.15 (3) Å^3^
*Z* = 2
Cu *K*α radiation
μ = 0.82 mm^−1^
*T* = 90 K
0.11 × 0.11 × 0.08 mm

#### Data collection

Bruker X8 Proteum diffractometer
Absorption correction: multi-scan (*SADABS* in *APEX2*; Bruker, 2006 ▶) *T*_min_ = 0.828, *T*_max_ = 0.938
13576 measured reflections
3294 independent reflections
3126 reflections with *I* > 2σ(*I*)
*R*_int_ = 0.035

#### Refinement

*R*[*F* ^2^ > 2σ(*F* ^2^)] = 0.037
*wR*(*F* ^2^) = 0.098
*S* = 1.07
3294 reflections
258 parameters
H-atom parameters constrained
Δρ_max_ = 0.37 e Å^−3^
Δρ_min_ = −0.41 e Å^−3^

### 

Data collection: *APEX2* (Bruker, 2006 ▶); cell refinement: *SAINT* (Bruker, 2006 ▶); data reduction: *SAINT*; program(s) used to solve structure: *SHELXS97* (Sheldrick, 2008 ▶); program(s) used to refine structure: *SHELXL97* (Sheldrick, 2008 ▶); molecular graphics: *XP* in *SHELXTL* (Sheldrick, 2008 ▶); software used to prepare material for publication: *SHELXL97* and local procedures.

## Supplementary Material

Crystal structure: contains datablocks global, I. DOI: 10.1107/S1600536809033881/hg2557sup1.cif

Structure factors: contains datablocks I. DOI: 10.1107/S1600536809033881/hg2557Isup2.hkl

Additional supplementary materials:  crystallographic information; 3D view; checkCIF report

## Figures and Tables

**Table 1:** Hydrogen-bond geometry (Å, °)

*D*—H⋯*A*	*D*—H	H⋯*A*	*D*⋯*A*	*D*—H⋯*A*
O2—H2⋯O1*S*	0.84	2.19	2.9579 (14)	152
O2—H2⋯O3^i^	0.84	2.58	3.2440 (12)	137
N3—H3*A*⋯O1^ii^	0.88	2.02	2.8898 (13)	169
N3—H3*B*⋯N2^iii^	0.88	2.06	2.9391 (15)	174
O1*S*—H1*S*⋯O3^i^	0.84	1.89	2.7048 (13)	162

Symmetry codes: (i) 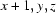; (ii) 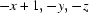; (iii) 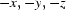.

**Fig. 1.**
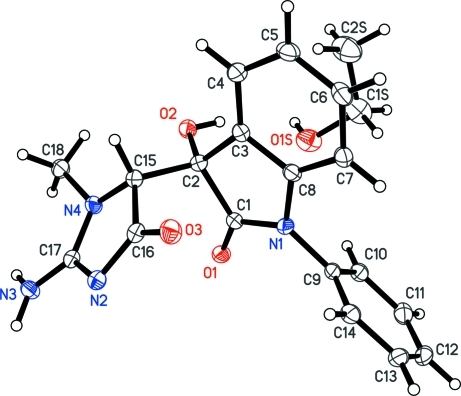
A view of the molecule with the atom numbering scheme. Displacement ellipsoids are drawn at the 50% probability level.

## supplementary crystallographic information

### Comment

1*H*-Indole-2,3-diones (isatins) are versatile molecules that display diverse biological activities, (Pandeya *et al.*, 2005), including anticancer activity. (Cane *et al.*, 2000 and Vine *et al.*, 2007). Based on the results of earlier work on radiosensitizers such as (*Z*)-2-(*N*-benzylindol-3-ylmethylene)quinuclidin-3-one and (*Z*)-(±)-2-(*N*-benzylindol-3-ylmethylene) quinuclidin-3-ol derivatives (Sekhar *et al.*, 2003; Sonar *et al.*, 2007), we have carried out the design, synthesis and structural analysis of a series of 3-(2-amino-1-methyl-4-oxo-4,5-dihydro-1*H*-imidazol-5-yl)-3- hydroxyindolin-2-one analogs with different substituents on the indole moiety. This X-ray analysis of the title compound was performed to confirm the stereochemistry of the molecule and to obtain detailed information on the conformation of the molecule, which may be useful in structure-activity relationship (SAR) analysis. The title compound was prepared by the aldol condensation of *N*-phenylindol-2,3-dione (*N* -phenyl isatin) with 2-amino- 1-methyl-1*H*-imidazol-4(5*H*)-one (creatinine) in the presence of sodium acetate in acetic acid under microwave irradiation. The compound was crystallized from ethyl alcohol. This aldol condensation reaction proceeds by the formation of the E-enolate, as per the Zimmerman-Traxler model (Zimmerman & Traxler, 1957), which favores anti products, and leads to the formation of a racemic compound (equimolar *RR* and S*S* enantiomers). The molecular structure and the atom-numbering scheme are shown in Fig.1. The isatin ring is planar (r.m.s. deviation = 0.00342 (10) Å) with bond distances and angles comparable with those previously reported for other isatin derivatives (Frolova *et al.*, 1988; Usman, *et al.*, 2002 and Penthala *et al.* 2009). The indole and creatinine moieties make a dihedral angle of 56.45 (4)°. Intermolecular N—H···O and O—H···N hydrogen bonds stabilize the crystal structure and form a supramolecular aggregation.

### Experimental

A mixture of *N*-phenyl isatin (1 mmol), creatinine (1.1 mmol) and sodium acetate (1.2 mmol) in acetic acid (1 ml) was irradiated in a domestic microwave oven for 30 sec with intermittent cooling every 5 sec. The reaction mixture was allowed to cool to room temperature, 10 ml of saturated sodium bicarbonate solution was added and the mixture was stirred for 10 minutes. The precipitate thus obtained was collected by filtration, washed with cold water and dried to afford the crude product. Crystallization from ethyl alcohol gave a white crystalline product of 3-(2-amino-1-methyl-4-oxo-4,5-dihydro-1*H*- imidazol-5-yl)-3-hydroxy-1-phenylindolin-2-one ethanolate which was suitable for X-ray analysis. ^1^H NMR (DMSO-d_6_): δ 3.25 (*s*, 3H), 4.22 (*s*, 1H), 6.62 (*s*, 1H, OH), 6.64–6.65 (*d*, J=2.4 Hz, 1H), 7.00–7.03 (*t*, J=7.5 Hz, 1H), 7.17–7.23 (*m*, 2H), 7.44–7.47 (*m*, 4H), 7.55–7.57 (*d*, J=7.5 Hz, 1H), 7.60 (*bs*, 2H, NH_2_) p.p.m.; ^13^C NMR (DMSO-d_6_): δ 33.02, 70.60, 76.26, 108.92, 122.74, 124.38, 126.95, 127.10, 128.13, 129.60, 129.86, 134.40, 143.93, 171.90, 174.09, 182.53 p.p.m..

### Refinement

H atoms were found in difference Fourier maps and subsequently placed in idealized positions with constrained distances of 0.98 Å (RCH_3_), 0.99 Å (R_2_CH_2_), 1.00 Å (R_3_CH), 0.95 Å (C_Ar_H), 0.84 Å (O—H), 0.88 Å (N—H), and with U_iso_(H) values set to either 1.2U_eq_ or 1.5U_eq_ (RCH_3_, OH) of the attached atom.

### Crystal data

**Table:**

C_18_H_16_N_4_O_3_·C_2_H_6_O	*Z* = 2
*M**_r_* = 382.42	*F*(000) = 404
Triclinic, *P*1	*D*_x_ = 1.391 Mg m^−^^3^
Hall symbol: -P 1	Cu *K*α radiation, λ = 1.54178 Å
*a* = 7.4912 (1) Å	Cell parameters from 9955 reflections
*b* = 11.0018 (2) Å	θ = 3.8–68.3°
*c* = 12.0835 (2) Å	µ = 0.82 mm^−^^1^
α = 78.152 (1)°	*T* = 90 K
β = 74.413 (1)°	Block, colourless
γ = 74.090 (1)°	0.11 × 0.11 × 0.08 mm
*V* = 913.15 (3) Å^3^	

### Data collection

**Table:**

Bruker X8 Proteum diffractometer	3294 independent reflections
Radiation source: fine-focus rotating anode	3126 reflections with *I* > 2σ(*I*)
graded multilayer optics	*R*_int_ = 0.035
Detector resolution: 5.6 pixels mm^-1^	θ_max_ = 68.3°, θ_min_ = 3.8°
φ and ω scans	*h* = −9→9
Absorption correction: multi-scan (*SADABS* in *APEX2*; Bruker, 2006)	*k* = −13→10
*T*_min_ = 0.828, *T*_max_ = 0.938	*l* = −14→14
13576 measured reflections	

### Refinement

**Table:**

Refinement on *F*^2^	Secondary atom site location: difference Fourier map
Least-squares matrix: full	Hydrogen site location: inferred from neighbouring sites
*R*[*F*^2^ > 2σ(*F*^2^)] = 0.037	H-atom parameters constrained
*wR*(*F*^2^) = 0.098	*w* = 1/[σ^2^(*F*_o_^2^) + (0.0501*P*)^2^ + 0.3708*P*] where*P* = (*F*_o_^2^ + 2*F*_c_^2^)/3
*S* = 1.07	(Δ/σ)_max_ < 0.001
3294 reflections	Δρ_max_ = 0.37 e Å^−^^3^
258 parameters	Δρ_min_ = −0.41 e Å^−^^3^
0 restraints	Extinction correction: *SHELXL97* (Sheldrick, 2008), Fc^*^=kFc[1+0.001xFc^2^λ^3^/sin(2θ)]^-1/4^
Primary atom site location: structure-invariant direct methods	Extinction coefficient: 0.0068 (7)

### Special details

**Table:**

Geometry. All e.s.d.'s (except the e.s.d. in the dihedral angle between two l.s. planes) are estimated using the full covariance matrix. The cell e.s.d.'s are taken into account individually in the estimation of e.s.d.'s in distances, angles and torsion angles; correlations between e.s.d.'s in cell parameters are only used when they are defined by crystal symmetry. An approximate (isotropic) treatment of cell e.s.d.'s is used for estimating e.s.d.'s involving l.s. planes.
Refinement. Refinement of *F*^2^ against ALL reflections. The weighted *R*-factor *wR* and goodness of fit *S* are based on *F*^2^, conventional *R*-factors *R* are based on *F*, with *F* set to zero for negative *F*^2^. The threshold expression of *F*^2^ > 2σ(*F*^2^) is used only for calculating *R*-factors(gt) *etc*. and is not relevant to the choice of reflections for refinement. *R*-factors based on *F*^2^ are statistically about twice as large as those based on *F*, and *R*- factors based on ALL data will be even larger.

### Fractional atomic coordinates and isotropic or equivalent isotropic displacement parameters (Å^2^)

**Table:**

	*x*	*y*	*z*	*U*_iso_*/*U*_eq_	
O1	0.37921 (12)	0.08545 (8)	0.23487 (7)	0.0188 (2)	
N1	0.16513 (14)	0.24923 (9)	0.32617 (8)	0.0159 (2)	
C1	0.29249 (17)	0.19721 (11)	0.23418 (10)	0.0161 (3)	
O2	0.50070 (12)	0.30752 (8)	0.07786 (7)	0.0202 (2)	
H2	0.5529	0.3158	0.1284	0.030*	
N2	0.03753 (14)	0.13607 (10)	0.04645 (8)	0.0174 (2)	
C2	0.30826 (17)	0.30455 (11)	0.12863 (10)	0.0173 (3)	
O3	−0.10708 (12)	0.31235 (9)	0.14096 (8)	0.0232 (2)	
N3	0.26263 (14)	0.00056 (10)	−0.07643 (9)	0.0185 (2)	
H3A	0.3780	−0.0201	−0.1202	0.022*	
H3B	0.1798	−0.0454	−0.0697	0.022*	
C3	0.18841 (17)	0.42116 (12)	0.18239 (10)	0.0178 (3)	
N4	0.33379 (14)	0.17486 (10)	−0.02748 (8)	0.0175 (2)	
C4	0.15762 (18)	0.54854 (12)	0.13580 (11)	0.0210 (3)	
H4	0.2148	0.5746	0.0573	0.025*	
C5	0.04026 (19)	0.63828 (12)	0.20688 (11)	0.0222 (3)	
H5	0.0180	0.7266	0.1768	0.027*	
C6	−0.04402 (18)	0.59920 (12)	0.32105 (11)	0.0212 (3)	
H6	−0.1239	0.6615	0.3680	0.025*	
C7	−0.01412 (17)	0.47040 (12)	0.36854 (10)	0.0183 (3)	
H7	−0.0728	0.4437	0.4465	0.022*	
C8	0.10411 (17)	0.38371 (11)	0.29745 (10)	0.0168 (3)	
C9	0.12524 (17)	0.18231 (11)	0.44190 (10)	0.0161 (3)	
C10	0.27258 (18)	0.12805 (11)	0.49893 (11)	0.0192 (3)	
H10	0.3994	0.1339	0.4615	0.023*	
C11	0.2316 (2)	0.06493 (12)	0.61182 (11)	0.0233 (3)	
H11	0.3312	0.0268	0.6516	0.028*	
C12	0.0459 (2)	0.05757 (12)	0.66629 (11)	0.0247 (3)	
H12	0.0185	0.0150	0.7435	0.030*	
C13	−0.10000 (19)	0.11217 (12)	0.60827 (11)	0.0233 (3)	
H13	−0.2271	0.1071	0.6460	0.028*	
C14	−0.06071 (18)	0.17416 (12)	0.49517 (11)	0.0195 (3)	
H14	−0.1599	0.2105	0.4548	0.023*	
C15	0.22403 (17)	0.28338 (11)	0.03230 (10)	0.0173 (3)	
H15	0.2094	0.3626	−0.0256	0.021*	
C16	0.03012 (17)	0.24701 (12)	0.08034 (10)	0.0175 (3)	
C17	0.21402 (17)	0.09987 (11)	−0.02122 (10)	0.0162 (3)	
C18	0.49912 (18)	0.18208 (12)	−0.12386 (10)	0.0207 (3)	
H18A	0.5813	0.0964	−0.1304	0.031*	
H18B	0.5707	0.2375	−0.1096	0.031*	
H18C	0.4562	0.2172	−0.1962	0.031*	
O1S	0.57472 (13)	0.29564 (10)	0.30873 (9)	0.0304 (3)	
H1S	0.6853	0.2952	0.2685	0.046*	
C1S	0.5142 (2)	0.39650 (15)	0.37674 (14)	0.0336 (3)	
H1S1	0.5991	0.3811	0.4309	0.040*	
H1S2	0.3837	0.3965	0.4237	0.040*	
C2S	0.5151 (2)	0.52356 (16)	0.30585 (16)	0.0413 (4)	
H2S1	0.6444	0.5248	0.2601	0.062*	
H2S2	0.4729	0.5894	0.3570	0.062*	
H2S3	0.4285	0.5405	0.2535	0.062*	

### Atomic displacement parameters (Å^2^)

**Table:**

	*U*^11^	*U*^22^	*U*^33^	*U*^12^	*U*^13^	*U*^23^
O1	0.0220 (4)	0.0146 (4)	0.0180 (4)	−0.0041 (3)	−0.0011 (3)	−0.0034 (3)
N1	0.0206 (5)	0.0124 (5)	0.0138 (5)	−0.0041 (4)	−0.0026 (4)	−0.0012 (4)
C1	0.0187 (6)	0.0160 (6)	0.0152 (6)	−0.0067 (5)	−0.0032 (5)	−0.0030 (4)
O2	0.0216 (5)	0.0249 (5)	0.0171 (4)	−0.0115 (4)	−0.0028 (3)	−0.0033 (3)
N2	0.0185 (5)	0.0188 (5)	0.0149 (5)	−0.0057 (4)	−0.0020 (4)	−0.0028 (4)
C2	0.0216 (6)	0.0160 (6)	0.0145 (6)	−0.0076 (5)	−0.0020 (5)	−0.0016 (5)
O3	0.0225 (5)	0.0246 (5)	0.0212 (5)	−0.0051 (4)	0.0000 (4)	−0.0080 (4)
N3	0.0177 (5)	0.0193 (5)	0.0199 (5)	−0.0067 (4)	−0.0011 (4)	−0.0068 (4)
C3	0.0231 (6)	0.0168 (6)	0.0160 (6)	−0.0068 (5)	−0.0061 (5)	−0.0026 (5)
N4	0.0191 (5)	0.0193 (5)	0.0151 (5)	−0.0075 (4)	0.0000 (4)	−0.0056 (4)
C4	0.0289 (7)	0.0183 (6)	0.0182 (6)	−0.0084 (5)	−0.0086 (5)	0.0002 (5)
C5	0.0306 (7)	0.0136 (6)	0.0253 (7)	−0.0054 (5)	−0.0126 (5)	−0.0003 (5)
C6	0.0253 (6)	0.0173 (6)	0.0234 (6)	−0.0017 (5)	−0.0096 (5)	−0.0068 (5)
C7	0.0220 (6)	0.0179 (6)	0.0165 (6)	−0.0048 (5)	−0.0061 (5)	−0.0033 (5)
C8	0.0215 (6)	0.0140 (6)	0.0173 (6)	−0.0058 (5)	−0.0071 (5)	−0.0017 (4)
C9	0.0236 (6)	0.0110 (5)	0.0131 (6)	−0.0043 (4)	−0.0017 (5)	−0.0028 (4)
C10	0.0230 (6)	0.0150 (6)	0.0188 (6)	−0.0033 (5)	−0.0032 (5)	−0.0042 (5)
C11	0.0337 (7)	0.0156 (6)	0.0194 (6)	−0.0009 (5)	−0.0089 (5)	−0.0024 (5)
C12	0.0409 (8)	0.0152 (6)	0.0154 (6)	−0.0078 (5)	−0.0014 (5)	−0.0011 (5)
C13	0.0280 (7)	0.0191 (6)	0.0206 (6)	−0.0099 (5)	0.0044 (5)	−0.0053 (5)
C14	0.0221 (6)	0.0162 (6)	0.0203 (6)	−0.0047 (5)	−0.0032 (5)	−0.0043 (5)
C15	0.0216 (6)	0.0165 (6)	0.0140 (6)	−0.0060 (5)	−0.0023 (5)	−0.0029 (4)
C16	0.0201 (6)	0.0190 (6)	0.0130 (5)	−0.0053 (5)	−0.0032 (5)	−0.0014 (4)
C17	0.0186 (6)	0.0179 (6)	0.0126 (5)	−0.0056 (5)	−0.0039 (4)	−0.0005 (4)
C18	0.0218 (6)	0.0246 (6)	0.0164 (6)	−0.0099 (5)	0.0011 (5)	−0.0054 (5)
O1S	0.0213 (5)	0.0303 (5)	0.0384 (6)	−0.0077 (4)	−0.0007 (4)	−0.0078 (4)
C1S	0.0254 (7)	0.0339 (8)	0.0426 (9)	−0.0049 (6)	−0.0067 (6)	−0.0115 (7)
C2S	0.0424 (9)	0.0358 (9)	0.0530 (10)	−0.0102 (7)	−0.0231 (8)	−0.0044 (7)

### Geometric parameters (Å, °)

**Table:**

O1—C1	1.2226 (15)	C7—C8	1.3770 (17)
N1—C1	1.3658 (15)	C7—H7	0.9500
N1—C8	1.4220 (15)	C9—C14	1.3876 (18)
N1—C9	1.4353 (15)	C9—C10	1.3886 (18)
C1—C2	1.5531 (16)	C10—C11	1.3929 (18)
O2—C2	1.4136 (15)	C10—H10	0.9500
O2—H2	0.8400	C11—C12	1.386 (2)
N2—C16	1.3477 (16)	C11—H11	0.9500
N2—C17	1.3562 (15)	C12—C13	1.388 (2)
C2—C3	1.5071 (17)	C12—H12	0.9500
C2—C15	1.5472 (16)	C13—C14	1.3895 (18)
O3—C16	1.2314 (15)	C13—H13	0.9500
N3—C17	1.3123 (16)	C14—H14	0.9500
N3—H3A	0.8800	C15—C16	1.5419 (17)
N3—H3B	0.8800	C15—H15	1.0000
C3—C4	1.3794 (17)	C18—H18A	0.9800
C3—C8	1.3918 (17)	C18—H18B	0.9800
N4—C17	1.3546 (16)	C18—H18C	0.9800
N4—C15	1.4560 (15)	O1S—C1S	1.4181 (18)
N4—C18	1.4621 (15)	O1S—H1S	0.8400
C4—C5	1.3972 (18)	C1S—C2S	1.483 (2)
C4—H4	0.9500	C1S—H1S1	0.9900
C5—C6	1.3870 (19)	C1S—H1S2	0.9900
C5—H5	0.9500	C2S—H2S1	0.9800
C6—C7	1.3960 (18)	C2S—H2S2	0.9800
C6—H6	0.9500	C2S—H2S3	0.9800
			
C1—N1—C8	110.74 (10)	C12—C11—C10	120.23 (12)
C1—N1—C9	124.57 (10)	C12—C11—H11	119.9
C8—N1—C9	123.74 (10)	C10—C11—H11	119.9
O1—C1—N1	125.87 (11)	C11—C12—C13	120.17 (12)
O1—C1—C2	125.91 (10)	C11—C12—H12	119.9
N1—C1—C2	108.21 (10)	C13—C12—H12	119.9
C2—O2—H2	109.5	C12—C13—C14	120.17 (12)
C16—N2—C17	106.75 (10)	C12—C13—H13	119.9
O2—C2—C3	115.27 (10)	C14—C13—H13	119.9
O2—C2—C15	106.63 (9)	C9—C14—C13	119.20 (12)
C3—C2—C15	110.59 (10)	C9—C14—H14	120.4
O2—C2—C1	111.04 (9)	C13—C14—H14	120.4
C3—C2—C1	101.86 (9)	N4—C15—C16	100.87 (9)
C15—C2—C1	111.52 (9)	N4—C15—C2	114.64 (10)
C17—N3—H3A	120.0	C16—C15—C2	112.68 (9)
C17—N3—H3B	120.0	N4—C15—H15	109.4
H3A—N3—H3B	120.0	C16—C15—H15	109.4
C4—C3—C8	120.49 (11)	C2—C15—H15	109.4
C4—C3—C2	130.40 (11)	O3—C16—N2	127.04 (11)
C8—C3—C2	109.10 (10)	O3—C16—C15	123.08 (11)
C17—N4—C15	107.56 (10)	N2—C16—C15	109.88 (10)
C17—N4—C18	122.36 (10)	N3—C17—N4	123.26 (11)
C15—N4—C18	122.80 (10)	N3—C17—N2	122.16 (11)
C3—C4—C5	118.36 (11)	N4—C17—N2	114.58 (10)
C3—C4—H4	120.8	N4—C18—H18A	109.5
C5—C4—H4	120.8	N4—C18—H18B	109.5
C6—C5—C4	120.40 (11)	H18A—C18—H18B	109.5
C6—C5—H5	119.8	N4—C18—H18C	109.5
C4—C5—H5	119.8	H18A—C18—H18C	109.5
C5—C6—C7	121.51 (12)	H18B—C18—H18C	109.5
C5—C6—H6	119.2	C1S—O1S—H1S	109.5
C7—C6—H6	119.2	O1S—C1S—C2S	112.92 (14)
C8—C7—C6	117.14 (11)	O1S—C1S—H1S1	109.0
C8—C7—H7	121.4	C2S—C1S—H1S1	109.0
C6—C7—H7	121.4	O1S—C1S—H1S2	109.0
C7—C8—C3	122.10 (11)	C2S—C1S—H1S2	109.0
C7—C8—N1	128.13 (11)	H1S1—C1S—H1S2	107.8
C3—C8—N1	109.73 (10)	C1S—C2S—H2S1	109.5
C14—C9—C10	121.20 (11)	C1S—C2S—H2S2	109.5
C14—C9—N1	119.23 (11)	H2S1—C2S—H2S2	109.5
C10—C9—N1	119.56 (11)	C1S—C2S—H2S3	109.5
C9—C10—C11	119.02 (12)	H2S1—C2S—H2S3	109.5
C9—C10—H10	120.5	H2S2—C2S—H2S3	109.5
C11—C10—H10	120.5		
			
C8—N1—C1—O1	174.88 (11)	C1—N1—C9—C10	57.36 (16)
C9—N1—C1—O1	5.70 (19)	C8—N1—C9—C10	−110.45 (13)
C8—N1—C1—C2	−5.06 (13)	C14—C9—C10—C11	−0.27 (18)
C9—N1—C1—C2	−174.24 (10)	N1—C9—C10—C11	179.09 (10)
O1—C1—C2—O2	−50.77 (15)	C9—C10—C11—C12	−0.49 (18)
N1—C1—C2—O2	129.18 (10)	C10—C11—C12—C13	0.53 (19)
O1—C1—C2—C3	−174.02 (12)	C11—C12—C13—C14	0.19 (19)
N1—C1—C2—C3	5.93 (12)	C10—C9—C14—C13	0.99 (18)
O1—C1—C2—C15	68.01 (15)	N1—C9—C14—C13	−178.38 (11)
N1—C1—C2—C15	−112.05 (11)	C12—C13—C14—C9	−0.94 (18)
O2—C2—C3—C4	53.97 (17)	C17—N4—C15—C16	4.68 (12)
C15—C2—C3—C4	−67.06 (16)	C18—N4—C15—C16	155.35 (10)
C1—C2—C3—C4	174.30 (13)	C17—N4—C15—C2	126.01 (10)
O2—C2—C3—C8	−125.10 (11)	C18—N4—C15—C2	−83.31 (13)
C15—C2—C3—C8	113.87 (11)	O2—C2—C15—N4	52.11 (12)
C1—C2—C3—C8	−4.77 (12)	C3—C2—C15—N4	178.14 (9)
C8—C3—C4—C5	0.01 (18)	C1—C2—C15—N4	−69.27 (13)
C2—C3—C4—C5	−178.97 (12)	O2—C2—C15—C16	166.73 (9)
C3—C4—C5—C6	−0.60 (19)	C3—C2—C15—C16	−67.24 (13)
C4—C5—C6—C7	0.32 (19)	C1—C2—C15—C16	45.36 (13)
C5—C6—C7—C8	0.56 (18)	C17—N2—C16—O3	178.51 (12)
C6—C7—C8—C3	−1.17 (18)	C17—N2—C16—C15	−1.87 (13)
C6—C7—C8—N1	176.39 (11)	N4—C15—C16—O3	177.90 (11)
C4—C3—C8—C7	0.91 (19)	C2—C15—C16—O3	55.19 (15)
C2—C3—C8—C7	−179.91 (11)	N4—C15—C16—N2	−1.74 (12)
C4—C3—C8—N1	−177.05 (11)	C2—C15—C16—N2	−124.45 (11)
C2—C3—C8—N1	2.13 (14)	C15—N4—C17—N3	172.92 (11)
C1—N1—C8—C7	−175.84 (12)	C18—N4—C17—N3	22.09 (18)
C9—N1—C8—C7	−6.55 (19)	C15—N4—C17—N2	−6.58 (13)
C1—N1—C8—C3	1.96 (14)	C18—N4—C17—N2	−157.41 (11)
C9—N1—C8—C3	171.25 (11)	C16—N2—C17—N3	−174.16 (11)
C1—N1—C9—C14	−123.26 (13)	C16—N2—C17—N4	5.35 (14)
C8—N1—C9—C14	68.92 (15)		

### Hydrogen-bond geometry (Å, °)

**Table:**

*D*—H···*A*	*D*—H	H···*A*	*D*···*A*	*D*—H···*A*
O2—H2···O1S	0.84	2.19	2.9579 (14)	152
O2—H2···O3^i^	0.84	2.58	3.2440 (12)	137
N3—H3A···O1^ii^	0.88	2.02	2.8898 (13)	169
N3—H3B···N2^iii^	0.88	2.06	2.9391 (15)	174
O1S—H1S···O3^i^	0.84	1.89	2.7048 (13)	162

Symmetry codes: (i) *x*+1, *y*, *z*; (ii) −*x*+1, −*y*, −*z*; (iii) −*x*, −*y*, −*z*.
